# Higher Orchiectomy Rates in Pre-pubescent Testicular Torsion: A Call for Earlier Recognition and Heightened Awareness

**DOI:** 10.7759/cureus.93512

**Published:** 2025-09-29

**Authors:** Lisa B Shields, Kahir Jawad, Eran Rosenberg

**Affiliations:** 1 Norton Neuroscience Institute, Norton Healthcare, Louisville, USA; 2 Norton Research Institute, Norton Healthcare, Louisville, USA; 3 Department of Pediatrics, University of Louisville School of Medicine, Louisville, USA; 4 Department of Pediatric Urology, Norton Healthcare, Louisville, USA

**Keywords:** age, orchiectomy, orchiopexy, pediatric urology, post-pubescent, pre-pubescent, testicular torsion

## Abstract

Background: Testicular torsion (TT) is a pediatric emergency that requires a prompt diagnosis and surgical intervention to increase the likelihood of testicular salvage. Pre-pubescent males have a heightened risk of undergoing an orchiectomy compared to post-pubescent males with TT. We sought to identify specific factors characteristic of pre-pubescent males compared to post-pubescent males.

Materials and methods: This retrospective analysis of a pediatric TT dataset over a 10-year period (January 1, 2015, to December 31, 2024) was conducted at a pediatric acute care children’s hospital in a metropolitan community. Males ages 1-20 years with TT were divided into two age groups: the pre-pubescent group ages 1-12 years and the post-pubescent group ages 13-20 years.

Results: A total of 286 patients were diagnosed with TT, including 208 (73%) post-pubescent males and 78 (27%) pre-pubescent males. There was a significant association between the pre- and post-pubescent patients and duration of TT symptoms (p=0.03). Of the 193 patients whose symptom duration was <24 hours, 148 (77%) were pre-pubescent compared to 45 (23%) who were post-pubescent. There was also a significant association between the two age groups and the type of surgery (p =0.001). The prevalence of orchiectomy in the younger group was 46.2% compared to 26.4% in the older group. Post-pubescent males had a 59% lower odds of undergoing an orchiectomy compared to pre-pubescent patients. The younger boys were 2.4 times more likely to have an orchiectomy compared to older boys.

Conclusions: Among children treated for TT, pre-pubescent males are at a higher risk for orchiectomy than post-pubescent males. Higher levels of awareness, as well as prompt evaluation and surgical exploration, are needed to increase the rates of salvageable testes.

## Introduction

Pediatric patients presenting with acute scrotal pain necessitate an urgent medical evaluation to confirm whether testicular torsion (TT) is the underlying etiology [1]. TT occurs when the testis rotates along its supporting ligaments, which results in testicular ischemia with potential loss of the testis [2]. TT is a urological emergency and requires hastened surgical intervention to offer the best chance of testicular viability. Testicular salvage rates are associated with the degrees of TT, as well as the duration of ischemia, with an optimal window of four to six hours between TT symptom onset and surgery [1]. Both duration of symptoms and time from presentation at the Emergency Department (ED) to surgery (referred to as the door to detorsion time (DTD)) are crucial factors in determining whether a testis can be salvaged [3-7]. The annual incidence of TT is 3.8 per 100,000 for males 18 years and younger, and it accounts for 10-15% of acute scrotal disease in children [8].

A bimodal distribution of TT exists in the pediatric population [2,9,10]. Most cases occur in the neonatal period to the first month of life and between the ages of 12-18 years, with 65% of TT cases comprising the latter age group [2,9,11]. While TT is more common in children and adolescents, it may also affect adult males [2]. Different features have been compared between pediatric and adult TT [12]. Barada et al. reported the impact of age on the time interval between the onset of TT symptoms and ED presentation, comparing pediatric (less than 18 years) and adult (older than 18 years) patients with TT [12]. A total of 44% of the younger patients required an orchiectomy compared to 8% of the adult group. The younger group comprised 90% of the total number of orchiectomies performed. These authors emphasize the importance of early evaluation of scrotal pain to increase testicular salvage [12]. Few studies have investigated the differences between pre- and post-pubescent males with TT [10,13-15].

Signs and symptoms of acute TT often include nausea, vomiting, acute scrotal pain, testicular tenderness, absent cremasteric reflex, scrotal swelling, and abnormal testicular lie within the scrotum [14]. However, younger patients with TT frequently do not present with these typical signs and symptoms. Due to vague symptoms often reported by children and adolescents, the diagnosis and treatment of TT is delayed, which results in a higher orchiectomy rate. In Goetz et al.’s study [14] of 91 patients with TT divided into the pre-pubescent group (ages 1-12 years, 33 patients) and the post-pubescent group (ages 13-18 years, 58 patients), pre-pubescent males were more likely to present with abdominal pain than post-pubescent males (27.3% vs 10.3%, respectively). Furthermore, patients who underwent an orchiectomy were more likely to have testicular swelling and abdominal pain than those who underwent an orchiopexy. The duration of symptoms is strongly associated with testicular salvage. Patients with a longer duration of symptoms are significantly more likely to undergo an orchiectomy. In Goetz et al.’s study, the duration of symptoms of the patients who underwent an orchiectomy was 45 hours compared to five hours in patients who underwent an orchiopexy (p < 0.001) [14].

In this report, we analyzed the features between the pre-pubescent and post-pubescent males with TT to determine whether there were differences in symptom duration, side of symptoms, DTD, type of surgery (orchiectomy versus orchiopexy), or degree of TT observed intraoperatively. We discuss the diverse characteristics between the pre- and post-pubescent patients. We also present the impediments to seeking prompt medical care for TT symptoms in pediatric patients, as well as public community campaigns to promote awareness of the urgent nature of TT.

## Materials and methods

Under an Institutional Review Board-approved protocol and conforming to the Declaration of Helsinki, we identified male children and adolescents ages 1-20 years with TT over a 10-year period (January 1, 2015, to December 31, 2024) at a pediatric acute care children’s hospital in a metropolitan community. The patients were divided into the pre-pubescent group (ages 1-12 years) and the post-pubescent group (ages 13-20 years). The age of 13 years was used as the cutoff between the two groups, as it represents the typical age of puberty onset according to the existing literature that utilized the World Health Organization age cutoff [14-16]. The first sign of pubertal development in males is an increase in testicular volume above 4 mL Tanner Genital stage 2, which occurs between 10 and 15 years [14,16,17]. Since TT typically occurs during puberty, it may be overlooked by providers in younger age groups. Therefore, we further subdivided the ages within the prepubertal group to determine if this was indeed the case. We performed a more detailed analysis of 3 categories of patient ages: (1) 1 to <10 years, (2) 10 to <14 years, and (3) 14-20 years.

Pediatric urologists at our acute care children's hospital obtained the medical history and performed a genitourinary physical examination in our institution’s ED. All patients underwent a Doppler scrotal ultrasound (US). Several characteristics were obtained, including the patients’ age, duration and side of symptoms, type of surgery (orchiectomy versus orchiopexy), DTD (duration between the start of ED triage and initiation of surgical anesthesia), and degree of TT observed intraoperatively.

Statistical analysis

Descriptive statistics were used to summarize the cohort characteristics. Continuous variables, assessed for normality using the Shapiro-Wilk test, were reported as median (Q1, Q3), while categorical variables were presented as frequencies and percentages. Univariate analyses were performed to evaluate associations between the pre- and post-pubescent groups: chi-square tests were used for categorical variables, including the age group, and Wilcoxon rank-sum tests were employed for continuous variables, such as age and DTD. Multivariable logistic regression models examined independent variables between the pre-pubescent and post-pubescent groups. All analyses were performed using a licensed version of SAS version 9.4 (SAS Institute Inc., Cary, NC) provided by Norton Children’s Research Institute, affiliated with the University of Louisville. All statistical analyses were conducted using SAS version 9.4.

## Results

Characteristics of pre- and post-pubescent patients with testicular torsion: univariate analysis

**Figure 1:**
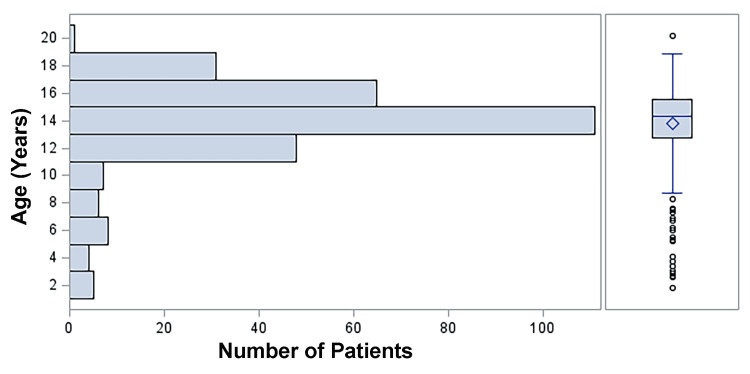
Distribution of Ages of the Pre- and Post-Pubescent Patients With Testicular Torsion

A total of 286 patients were diagnosed with TT over the duration of this study (Table 1). The median age was 14.3 years (range: 21 months to 20 years; Q1 (25th percentile): 12.8 years, Q3 (75th percentile): 15.6 years), with 208 (73%) post-pubescent patients and 78 (27%) pre-pubescent patients. Figure 1 depicts the distribution of patient ages. The median duration of symptoms was nine hours (ranging between 30 minutes and 336 hours): six hours with a viable testis (underwent an orchiopexy) and 48 hours with a nonviable testis (underwent an orchiectomy) (p < 0.001). For a symptom duration less than 24 hours, 163 (84%) patients underwent an orchiopexy compared to 30 (16%) who had an orchiectomy. For a symptom duration of 24 hours or greater, 61 (66%) patients underwent an orchiectomy versus 32 (34%) who had an orchiopexy.

**Table 1:** Characteristics of Testicular Torsion Divided by Pre- and Post-Pubescent Patients ^a^ p-value of the Wilcoxon rank sum test; ^b^ p-value of the chi-square test All statistical analyses were conducted using SAS version 9.4 (SAS Institute Inc., Cary, NC).

Variables	Total	Age < 13 yrs	Age ≥ 13 yrs	Test Statistics Value	P-value
Overall	N =286	78 (27%)	208 (73%)		
Duration of symptoms (hrs), median (Q1, Q3)	9 (4, 48)	16 (6, 48)	8 (4, 34)	Z = 2.37	0.018^a^
Duration of symptoms, n (%)				c^2^ = 10.00	0.075^b^
0 - < 6 hrs	99 (35%)	17 (17%)	82 (83%)		
6 hrs - < 12 hrs	54 (19%)	17 (31%)	37 (69%)		
12 hrs - < 18 hrs	27 (9%)	7 (26%)	20 (74%)		
18 hrs - < 24 hrs	13 (5%)	4 (31%)	9 (69%)		
24 hrs - < 48 hrs	20 (7%)	9 (45%)	11 (55%)		
≥ 48 hrs	73 (26%)	24 (33%)	49 (67%)		
Duration of symptoms, n (%)				c^2^ = 4.68	0.030^b^
< 24 hrs	193 (67%)	45 (23%)	148 (77%)		
≥ 24 hrs	93 (33%)	33 (35%)	60 (65%)		
Door To Detorsion time (mins), median	147 minutes (Range: 25-668 minutes)	153 minutes (Range: 40-343 minutes)	142 minutes (Range: 25-668 minutes)	Z = 0.76	0.448^a^
Degree of testicular torsion, median (Q1, Q3)	360 (180, 720)	360 (180, 720)	360 (180, 720)	Z = 0.115	0.909^a^
Side, n (%)				c^2^ = 2.39	0.122^b^
Left	135 (47%)	31 (23%)	104 (77%)		
Right	151 (53%)	47 (31%)	104 (69%)		
Type of Surgery, n (%)				c^2^ = 10.16	0.001^b^
Orchiopexy	195 (68%)	42 (22%)	153 (78%)		
Orchiectomy	91 (32%)	36 (40%)	55 (60%)		

A chi-square test revealed a significant association between the pre- and post-pubescent patients and duration of TT symptoms when divided into two time categories (≥24 hours versus <24 hours) (p = 0.030). Of the 193 patients whose duration of symptoms was <24 hours, 148 (77%) were post-pubescent compared to 45 (23%) who were pre-pubescent. There was also a significant association between the pre- and post-pubescent groups and the type of surgery (orchiectomy versus orchiopexy) (p = 0.001). Of the 195 patients who underwent an orchiopexy, 153 (78%) were post-pubescent compared to 42 (22%) who were pre-pubescent. The prevalence of orchiectomy in the pre-pubescent group was 46.2% compared to 26.4% in the post-pubescent group. The median duration of symptoms was significantly different between the pre- and post-pubescent groups, as indicated by the Wilcoxon rank sum test (p = 0.018). The overall median duration of symptoms was nine hours (range: 30 minutes to 336 hours): 16 (1-336 hours) in the pre-pubescent group versus eight (30 minutes to 168 hours) in the post-pubescent group (p = 0.018). 

When the patients were divided into three age groups (1 - <10 years, 10 - <14 years, and 14-20 years), there was a significant association between the three age groups and duration of symptoms (≥24 versus <24 hours) (p = 0.003), type of surgery (orchiectomy versus orchiopexy) (p < 0.001), the side of TT (p = 0.048), and symptom duration (0 to <6 hours, 6 to <12 hours, 12 to <18 hours, 18 to <24 hours, 24 to <48 hours, and ≥48 hours) (p = 0.026) (Table 2). The median duration of symptoms was significantly different between the three age groups, as indicated by the Wilcoxon rank sum test (p = <0.001).

**Table 2:** Patient Characteristics of Testicular Torsion by Age Groups ^a^ p-value of the Kruskal-Wallis test; ^b^ p-value of the chi-square test;^ c^ p-value of Fisher's exact test All statistical analyses were conducted using SAS version 9.4 (SAS Institute Inc., Cary, NC).

Variables	Total	Age 1-<10 yrs	Age 10-<14	Age 14-20	Test Statistics Value	P-value
Overall	N = 286	25 (8.7%)	96 (33.6%)	165 (57.7%)		
Duration of symptoms (hrs), median (Q1, Q3)	9 (4, 48)	48 (11, 72)	12 (5, 48)	6 (4, 24)	c^2^(2) = 16.14	< 0.001^a^
Duration of symptoms, n (%)					-	0.027^c^
0 - < 6 hrs	99 (35%)	3 (12%)	26 (27%)	70 (42%)		
6 hrs - < 12 hrs	54 (19%)	4 (16%)	18 (19%)	32 (19%)		
12 hrs - < 18 hrs	27 (9%)	2 (8%)	12 (13%)	13 (8%)		
18 hrs - < 24 hrs	13 (5%)	1 (4%)	6 (6%)	6 (4%)		
24 hrs - < 48 hrs	20 (7%)	2 (8%)	8 (8%)	10 (6%)		
≥ 48 hrs	73 (26%)	13 (52%)	26 (27%)	34 (21%)		
Duration of symptoms, n (%)					c^2^ = 11.55	0.003^b^
< 24 hrs	193 (67%)	10 (40%)	62 (65%)	121 (73%)		
≥ 24 hrs	93 (33%)	15 (60%)	34 (35%)	44 (27%)		
Door to detorsion (hrs), median (Q1, Q3)	2 (2, 3)	3 (2, 4)	2 (2, 3)	2 (2, 3)	c^2^(2) = 3.02	0.221^a^
Degree of torsion, median (Q1, Q3)	360 (180, 720)	360 (360, 720)	360 (180, 720)	360 (180, 720)	c^2^(2) = 0.52	0.771^a^
Side, n (%)					c^2^ = 6.06	0.048^b^
L	135 (47%)	6 (24%)	46 (48%)	83 (50%)		
R	151 (53%)	19 (76%)	50 (52%)	82 (50%)		
Type of Surgery, n (%)					c^2^ = 20.02	< 0.001^b^
Orchiopexy	195 (68%)	8 (32%)	62 (65%)	125 (76%)		
Orchiectomy	91 (32%)	17 (68%)	34 (35%)	40 (24%)		

Characteristics of pre- and post-pubescent patients with testicular torsion: multivariate analysis

Post-pubescent patients had a 59% lower odds of undergoing an orchiectomy compared to pre-pubescent patients, after adjusting for DTD (adjusted OR = 0.41 (95% CI: 0.23-0.71, p=0.002)) (Table 3). Pre-pubescent boys were 2.4 times more likely to have an orchiectomy compared to post-pubescent boys. For each additional hour of delay from ED triage to surgery, the odds of an orchiectomy increased by 43%, adjusting for age. Thus, a three-hour delay increased the odds of an orchiectomy by 3.3 (1.43³ ≈ 2.92).

**Table 3:** Factors Predictive of Orchiectomy in Multivariate Logistic Regression Comparing Pre- and Pre-Postpubescent Age Groups DTD: door to detorsion time All statistical analyses were conducted using SAS version 9.4 (SAS Institute Inc., Cary, NC).

Variables	Adjusted OR	95% CI	P-value	Test Statistic Numerical Value
Age group (≥13 yrs vs <13 yrs)	0.41	(0.23 - 0.71)	0.002	Wald c^2^ = 10.10
DTD (hrs)	1.43	(1.18 - 1.74)	< 0.001	Wald c^2^ = 13.17

When the patients were divided into three age groups, the youngest age group (1-10 years) had a 6.5 times higher odds of undergoing an orchiectomy compared to the oldest age group (≥14 years), adjusting for DTD (Table 4). Children in the 10 to <14 years age group had twice as high odds of undergoing an orchiectomy compared to the oldest age group (≥14 years), adjusting for DTD.

**Table 4:** Factors Predictive of Orchiectomy in Multivariate Logistic Regression Comparing Three Age Groups DTD: door to detorsion time All statistical analyses were conducted using SAS version 9.4 (SAS Institute Inc., Cary, NC).

Variables	Adjusted OR	95% CI	P-value	Test Statistic Numerical Value
Age group: <10 yrs vs ≥14 yrs	6.47	(2.54 - 16.51)	< 0.001	Wald c^2^ = 15.27
Age group: 10-<14 yrs vs ≥14 yrs	1.95	(1.10 - 3.43)	0.022	Wald c^2^ = 5.27
DTD (hrs)	1.43	(1.17 - 1.74)	< 0.001	Wald c^2^ = 12.69

## Discussion

A paucity of studies have evaluated the different characteristics of TT among pre- and post-pubescent patients [10,13-15]. In Goetz et al.’s study of 91 patients with TT divided into the pre-pubescent group (ages 1-12 years, 33 patients) and the post-pubescent group (ages 13-18 years, 58 patients), the overall orchiectomy rate was 30.8% [14]. More pre-pubescent males underwent an orchiectomy than post-pubescent males (42.4% versus 24.1%, respectively). Pre-pubescent males were also more likely to have a delayed presentation and diagnosis with a longer DTD, resulting in a delayed surgical intervention. The risk of orchiectomy decreased by 14% per one-year increase in age, and a steady decline in the proportion of patients undergoing orchiectomy was observed from 1 to 12 years of age. In Cost et al.’s analysis of national orchiopexy versus orchiectomy rates, a higher orchiectomy rate was noted in patients ages one to nine versus 10 years and older [13]. This finding was confirmed by Zhao et al. in their assessment of pediatric TT epidemiology using a national database [10]. These authors reported that the odds ratio for an orchiectomy was the highest for children in the youngest age quartile (younger than 10 years old) [10]. In our previous six-year study of 140 patients with TT comparing pre- and post-pubescent males, pre-pubescent boys had a longer symptom duration (18 versus 8 hours) and underwent more orchiectomies (58% versus 32% of patients) compared with post-pubescent boys [15]. The risk of orchiectomy decreased by 15% per one-year increase in age. Post-pubescent boys were 63% less likely to receive an orchiectomy versus an orchiopexy compared with pre-pubescent boys.

We have greatly expanded our previous investigation of the different features between pre- and post-pubescent males by augmenting the study duration from six to 10 years and doubling the number of patients from 140 to 286. This increased duration of evaluation of pediatric TT and a substantially increased number of patients allow us to confirm the findings of our previous study and add to the existing literature by presenting the largest number of patients with TT over the longest period of time. Additionally, in the current study, we have performed a more detailed analysis of the specific ages in the pediatric age group with TT. Instead of only dividing the patients into pre- and post-pubescent patients with TT, we have divided the patients into three age groups to assess whether the youngest patients were more likely to undergo an orchiectomy. Our results confirmed that the youngest age group had 6.5 times higher odds of undergoing an orchiectomy compared to the oldest age group. These findings are a valuable contribution to the existing literature as they encourage the youngest patients with TT to promptly undergo assessment in the ED to increase the likelihood of testicular salvage.

Our present study concurs with those in the literature regarding the increased likelihood of pre-pubescent males to have a longer symptom duration and undergo more orchiectomies compared to post-pubescent males. Post-pubescent patients had a 59% lower odds of undergoing an orchiectomy compared to pre-pubescent males after adjusting for DTD, indicating that the former are 2.4 times more likely to have a nonviable testis. This may be due to a delayed diagnosis in the younger cohort, as they may have a greater difficulty communicating their pain [18]. The salvage rate is higher in the post-pubescent group, which may be attributed to a faster recognition of symptoms. For each additional hour of delay from ED arrival to surgery, the odds of an orchiectomy increased by 43%, independent of age. In this respect, a three-hour delay increased the odds of an orchiectomy by 3.3. A prolonged DTD significantly reduced testicular salvageability, confirming the need for an expedited ED evaluation, prompt US, and accelerated arrival to the operating room.

Several factors may account for the higher orchiectomy rates in pre-pubescent males compared to post-pubescent patients. Younger boys often present with non-scrotal symptoms, such as abdominal pain, nausea and vomiting, and general irritability or lethargy. These nonspecific symptoms may result in a misdiagnosis, such as gastroenteritis or appendicitis, which may curtail prompt diagnosis and treatment of TT [14]. Pre-pubescent males may have difficulty clearly describing the location or severity of pain due to either a lack of vocabulary or embarrassment, which may lead to underreporting or misunderstanding of symptoms [18]. A rat model has suggested that the smaller and less vascularized testes of prepubertal rats may have a shorter tolerance window to ischemia [19]. Pre-pubertal testicular cells have a lower mitochondrial function and are more vulnerable to oxidative stress.

Despite being one of the most common emergencies in pediatric urology, knowledge of the urgent nature of this condition is rare among children/adolescents and their parents [20-22]. In Friedman et al.’s study of a survey of 479 urology parents and 59 ENT parents, only 34% of urology parents had heard of TT, most commonly through friends, relatives, or knowing someone with TT [21]. Only 17% were informed by pediatricians. Only 13% of parents of boys had spoken with their sons about TT. These authors admit that knowledge on TT is lacking and stress the need for standardized, effective education about TT for both parents and their male children/adolescents [21]. In MacDonald et al.’s study of interviews with males 11-19 years of age, the participants revealed several factors for the delay in ED presentation including not engaging in healthcare services independently of their parents, lacking knowledge about testicular pathology (both participants and their parents), participants not wanting to raise a false alarm, and families not wanting to encumber the healthcare system and would prefer to delay medical care until the next day [22]. Similarly, these authors recommend a testicular health education initiative for male pediatric patients and educating parents that certain medical conditions, such as TT, should not be viewed as “watch and wait” [22]. In Alyami et al.’s study of 200 parents (100 in the pediatric urology clinical and 100 in the general pediatric clinic), 19% of pediatric urology clinical parents and 14% of general pediatric clinic parents were aware of TT [20]. These authors emphasize that parents should be more aware of TT and their sons’ complaints of scrotal pain due to potential negative repercussions, such as testicular loss [20]. Additionally, an emergency physician may be less likely to consider TT in pre-pubescent males if specific genital complaints are not broached [23]. Continuing education in promoting awareness of TT in the ED is vital to decrease the risk of missed and delayed diagnosis of TT [23].

Awareness of the symptoms of TT and the importance of prompt medical intervention is of utmost importance for the general public. In our study, patients with a shorter duration of symptoms were statistically more likely to undergo an orchiopexy (six hours) versus an orchiectomy (48 hours). Furthermore, for a symptom duration less than 24 hours, 84% of patients underwent an orchiopexy compared to 16% who had an orchiectomy. These findings confirm the importance of timely ED arrival following suspicion of TT to reduce the likelihood of undergoing an orchiectomy. Awareness campaigns have been initiated to encourage recognition of suspected TT [24]. The Getting It Right First Time (GIRFT) expert working group convened a multidisciplinary group of urologists, pediatric surgeons, general surgeons, anesthesiologists, emergency care physicians, and radiologists to develop a national consensus pathway for best practices in managing acute testicular pain. This team was spurred by the findings of the report Twist and Shout, which reviewed the care provided to 827 children and adolescents with TT between 2021 and 2022 [24]. This report revealed that TT was not recognized by 66% of patients and 36% of parents/caregivers, and that care is delayed, which increases the likelihood of an orchiectomy. The GIRFT team acknowledged that the lack of awareness of TT among children and adolescents may be due to the private and sensitive nature of TT [24]. They recommended that education about TT should be provided in the school system so that boys will recognize the symptoms of acute testicular pain and be prepared to broach the subject with an adult (teacher, parent, or other caregivers).

Strengths and limitations

The strength of the current study is the large number of children and adolescents with TT over a 10-year duration. By dividing the pediatric patients with TT into pre- and post-pubescent groups, we were able to analyze the characteristic findings of each group, which permits a targeted approach to managing this urological emergency. Another strength is that we performed a more detailed age analysis following the initial pre- and post-pubescent assessment, revealing that there was a higher likelihood of an orchiectomy the younger the patient was. A limitation of this study includes its retrospective nature. Another limitation is recall bias, in that patients may not recall the exact duration of their symptoms. Additionally, examiner bias may have been present as several pediatric urologists at our acute care children’s hospital evaluated the pediatric patients with TT and performed the surgery (orchiectomy versus orchiopexy).

## Conclusions

This study of 286 pediatric cases of TT over a 10-year period compared males in the pre-pubescent group (ages 1-12 years) and post-pubescent group (ages 13-20 years) to identify distinct factors of pre-pubescent males compared to post-pubescent males. Pre-pubescent males are more likely to have a longer duration of TT symptoms and a higher orchiectomy rate compared to post-pubescent males. A delayed DTD also increases the likelihood of an orchiectomy, reflecting the need for an expedited assessment of all pediatric patients suspected of having TT from ED arrival to surgery. A high index of suspicion for TT is needed in the ED for pre-pubescent boys who present with indistinct symptoms. A public health initiative targeting pre-pubescent males and their families, school system, and pediatrician is warranted to educate about the symptoms of TT and underscore the importance of urgently seeking medical care.

## References

[1] Bowlin PR, Gatti JM, Murphy JP (2017). Pediatric testicular torsion. Surg Clin North Am.

[2] Lacy A, Smith A, Koyfman A, Long B (2023). High risk and low prevalence diseases: testicular torsion. Am J Emerg Med.

[3] Gold DD, Lorber A, Levine H (2019). Door to detorsion time determines testicular survival. Urology.

[4] Howe AS, Vasudevan V, Kongnyuy M (2017). Degree of twisting and duration of symptoms are prognostic factors of testis salvage during episodes of testicular torsion. Transl Androl Urol.

[5] Mellick LB, Sinex JE, Gibson RW, Mears K (2019). A systematic review of testicle survival time after a torsion event. Pediatr Emerg Care.

[6] Morin OA, Carr MG, Holcombe JM, Bhattacharya SD (2019). Optimal predictor of gonadal viability in testicular torsion: time to treat versus duration of symptoms. J Surg Res.

[7] Steeman A, Ngatchou W, Ramadan AS (2022). Impact of treatment delays on outcome of acute testicular torsion: a 15-year retrospective study. Acta Chir Belg.

[8] Sharp VJ, Kieran K, Arlen AM (2013). Testicular torsion: diagnosis, evaluation, and management. Am Fam Physician.

[9] Appelbaum R, Azari S, Clement M, Browne M (2019). Testicular torsion: the unexpected terrible twos, a unique case report. J Pediatr Surg Case Rep.

[10] Zhao LC, Lautz TB, Meeks JJ, Maizels M (2011). Pediatric testicular torsion epidemiology using a national database: incidence, risk of orchiectomy and possible measures toward improving the quality of care. J Urol.

[11] Onwuasoanya UE (2021). Atypical presentation of testicular torsion: a case series. Afr J Urol.

[12] Barada JH, Weingarten JL, Cromie WJ (1989). Testicular salvage and age-related delay in the presentation of testicular torsion. J Urol.

[13] Cost NG, Bush NC, Barber TD, Huang R, Baker LA (2011). Pediatric testicular torsion: demographics of national orchiopexy versus orchiectomy rates. J Urol.

[14] Goetz J, Roewe R, Doolittle J (2019). A comparison of clinical outcomes of acute testicular torsion between prepubertal and postpubertal males. J Pediatr Urol.

[15] Shields LBE, Daniels MW, Peppas DS, Rosenberg E (2023). Differences in clinical characteristics between prepubescent and postpubescent males with testicular torsion. Clin Pediatr (Phila).

[16] World Health Organization (2010). Sexual maturity rating (Tanner staging) in adolescents. Antiretroviral Therapy for HIV Infection In Infants and Children: Towards Universal Access.

[17] Emmanuel M, Bokor BR (2025). Tanner stages. StatPearls.

[18] Shields LBE, Daniels MW, Peppas DS, Rosenberg E (2021). Testicular torsion in patients with intellectual and developmental disabilities. Glob Pediatr Health.

[19] Almodhen F, He X, Loutochin O, Jednak R, Capolicchio JP, El-Sherbiny MT (2011). Protective role of hypothermia on ischemia of prepubertal rodent testicle. Urology.

[20] Alyami FA, Modahi NH, Alharbi AM, Alkhelaif AA, Alhazmi H, Trbay MS, Neel KF (2019). Parents' awareness and knowledge of testicular torsion: a cross-sectional study. Urol Ann.

[21] Friedman AA, Ahmed H, Gitlin JS, Palmer LS (2016). Standardized education and parental awareness are lacking for testicular torsion. J Pediatr Urol.

[22] MacDonald C, Burton M, Carachi R, O'Toole S (2021). Why adolescents delay with presentation to hospital with acute testicular pain: a qualitative study. J Pediatr Surg.

[23] Cheng EM, Chui JN, Crowe M, Cooke A (2022). Improving testicular examinations on paediatric patients in the emergency department: a quality improvement study to improve early diagnosis of testicular torsion. Asian J Urol.

[24] Getting It Right First Time (2025). Getting It Right First Time: Expert working group develops national consensus pathway for suspected testicular torsion. https://gettingitrightfirsttime.co.uk/expert-working-group-develops-national-consensus-pathway-for-suspected-testicular-torsion/.

